# Genome-wide association study analysis of single nucleotide variants in *L. infantum* associated with IL-6 inflammatory response in visceral leishmaniasis

**DOI:** 10.1017/S0031182024001598

**Published:** 2025-07

**Authors:** Amanda Miranda da Silva, Kátia Silene Sousa Carvalho, Caio Andrey Bezerra Januário, Raquel Gomes de Sena Carneiro Caldas, Bianka Lopes da Silva Paulino, Débora Cavalcante Braz, Dorcas Lamounier Costa, Gabriel da Luz Wallau, Wilson Jose da Silva Junior, Carlos Henrique Nery Costa

**Affiliations:** 1 Postgraduate Program in Biotechnology, Northeast Network of Biotechnology, Federal University of Piauí, Piauí, Brazil; 2 Leishmaniasis Research Laboratory – LabLeish, Piauí, Brazil; 3 Postgraduate Program in Genetics and Molecular Biology, Federal University of Pernambuco – UFPE, Pernambuco, Brazil; 4 Postgraduate Program in Biological Sciences, Federal University of Pernambuco – UFPE, Pernambuco, Brazil; 5 Pharmacy Course, Federal University of Piauí, Piauí, Brazil; 6 Natan Portella Institute of Tropical Diseases, Piauí, Brazil; 7 Center for Intelligence on Emerging and Neglected Tropical Diseases – CIATEN, Piauí, Brazil; 8 Department of Entomology, Instituto Aggeu Magalhães, Fundação Oswaldo Cruz – Fiocruz, Pernambuco, Brazil

**Keywords:** genome-wide association study, genomics, interleukin-6, *Leishmania infantum*, visceral leishmaniasis

## Abstract

Elevated levels of IL-6 in plasma are associated with the severity of visceral leishmaniasis (VL). The clinical manifestations of VL vary among patients, influenced by host factors and the virulence of the *Leishmania infantum* parasite. Considering that severe VL may result from an exaggerated inflammatory response, this study investigated whether IL-6 could serve as a biomarker to identify pro-inflammatory virulence factors. We conducted a genome-wide association study (GWAS) analysis on *L. infantum* isolates from patients with VL, whose IL-6 concentrations were measured. The analysis revealed that the relationship between IL-6 levels and clinical outcomes (survival *vs* mortality) had an area under the curve (AUC) of 0.67 (95% CI 0.52–0.81). A cut-off of 391.7 pg mL^−1^ for IL-6 was established to conduct a logistic regression analysis. We identified 10 029 single nucleotide variants (SNVs) across 62 genomes, resulting in 6,948 SNVs after filtering, of which 6,341 are located in protein-coding regions. The association analysis with PLINK identified 722 variants, of which 35 showed significant associations, with odds ratios ≥3.3, primarily in coding regions. These findings demonstrate that IL-6 levels tended to be associated with the fatal outcome of VL and highlight 35 novel genetic variants that could serve as potential biomarkers for prognosis. Further research into the biological role of these variants may lead to new therapeutic targets and improve the clinical management of VL, especially in identifying high-risk patients.

## Graphical abstract

**Figure:**
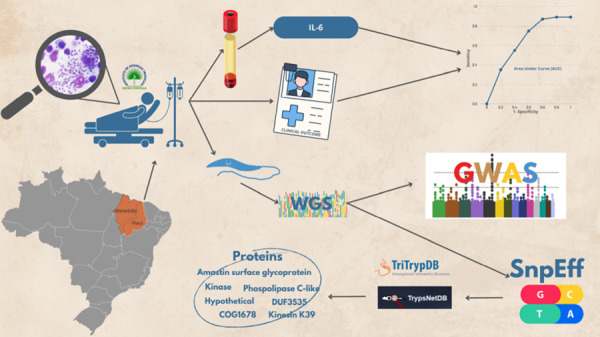

## Introduction

Visceral leishmaniasis (VL), or kala-azar, is the most severe form of leishmaniasis (Ruiz-Postigo, 2021). In 2020, VL was endemic in approximately 79 countries, with 1,834 cases reported in 13 Latin American countries, including Brazil, which accounted for 92% of the region's cases (OPAS, 2023). If left untreated, the disease can be fatal, especially in infants and the elderly. VL is caused by different species of protozoa from the genus *Leishmania*, which exhibit opportunistic behaviour, usually associated with social factors and primarily affecting the most vulnerable age groups, such as patients with comorbidities and/or immunosuppressive diseases (Burza *et al*., 2018).

The clinical characterization and cure of the disease are associated with the development of an effective and balanced immune response. The characteristics of the immune responses attributed to *Leishmania* infections vary among affected individuals, resulting in different clinical outcomes. These discrepancies arise from factors such as the host's immune profile, the species of *Leishmania*, exposure to the parasite, coinfections and other factors (Liese *et al*., 2008; Ribeiro *et al*., 2020).

The innate and adaptive immune response is part of a complex attempt by the organism to resist *Leishmania*. The cell-mediated immune response plays a crucial role in both clinical cure and disease progression. Effector cells, such as monocytes and macrophages, are essential for the regression or progression of the infection. These cells act as antigen-presenting cells and are involved in the early responses of the innate immune system, requiring mechanisms that modulate the activation of the inflammatory response. This occurs through the production of cytokines such as TNF-*α*, NO and reactive oxygen intermediates, which function to eliminate the parasite. However, cytokines related to the Th2 pathway, such as IL-4, IL-10 and TGF-*β*, are associated with the maintenance and survival of the parasites within the cells (Costa *et al*., 2013; Dayakar *et al*., 2019; Samant *et al*., 2021).

Evidence in humans demonstrates that the uncontrolled increase in the production of inflammatory cytokines in VL significantly contributes to the pathogenesis of the disease. Several studies show elevated serum levels of cytokines such as IL-4, IL-6, IL-12, IFN-*γ* and TNF-*α* during the active phase of the disease compared to asymptomatic infection (Peruhype-Magalhães *et al*., 2005; Peruhype-Magalhães *et al*., 2006; Costa *et al*., 2012). IL-6 plays a crucial role in the progression of VL, exhibiting various effects, such as inducing immunosuppression in the liver of the infected host, increasing hypergammaglobulinemia, and inhibiting TNF-*α* production during the early stage of infection (De Lima *et al*., 2007; Murray, 2008; Samant *et al*., 2021). High levels of IL-6 in patients with VL are associated with the severity of the disease, with greater concentrations of inflammatory cytokines compared to less severe cases. Furthermore, IL-6 is related to symptoms such as haemorrhages, vomiting and changes in laboratory tests, as well as markers of disseminated intravascular coagulation. Therefore, disease progression is closely linked to the dysregulation of the inflammatory response, which, in turn, contributes to the development of systemic inflammatory syndrome (Costa *et al*., 2010, 2013; Ribeiro *et al*., 2020; Guedes *et al*., 2022).

The host's genetics is undoubtedly a crucial factor in susceptibility to VL, as evidenced by genetic influence on infections by *L. infantum* and *L. donovani* (Blackwell *et al*., 2009). The genetic diversity of the *L. infantum* parasite is fundamental to the mortality associated with leishmaniasis, as demonstrated in the study by Grace *et al*. (2022). Different isolates of this parasite exhibit variations in their virulence, which implies that the risk of mortality may vary depending on the isolate responsible for the infection (Grace *et al*., 2022). Advances in leishmaniasis research have been significantly accelerated by the availability of genomic data, which are essential for investigations using genomic, transcriptomic and proteomic approaches (Cruz and Freitas-Castro, 2019). The first genome of *L. infantum* was sequenced and published in 2007, marking a milestone that enabled various investigations through DNA sequencing technology. This innovation facilitated the exploration of the genetic structure of the parasite and resulted in significant discoveries about the species (Peacock *et al*., 2007). The *L. infantum* genome is approximately 32 Mb, distributed across 36 chromosomes, with a total of 32 802 969 base pairs (González-de *et al*., 2017). The assessment of genetic diversity within the species cannot be conducted based on a single genome, making the study of other genomes from specific parasite populations a valuable tool (Hall, 2007).

Genome-wide association studies (GWAS) are powerful tools for connecting a phenotype to its underlying genetic basis. This method offers a hypothesis-free approach, systematically testing hundreds of thousands of variants across the genome without the need for prior knowledge about the location of causal variants. GWAS investigates small variations, known as single nucleotide variants (SNVs), throughout the genome that occur more frequently in organisms with a specific phenotype than in organisms not related to that phenotype (Korte and Farlow, 2013; Simon *et al*., 2016; Marigorta *et al*., 2018). In this context, the importance of genomic annotation databases, such as Tritryp and TrypsNetDB, becomes evident, as they provide crucial information about the location and functionality of these variants in *Leishmania*. These resources enable researchers to integrate variant data with information about genes, biological functions and phenotypic characteristics, facilitating the identification of potential causal variants. Furthermore, these databases assist in validating results and formulating hypotheses about the functional role of identified variants, promoting a deeper understanding of the genetic basis of complex phenotypes (Gazestani *et al*., 2017; Shanmugasundram *et al*., 2023).

Thus, based on the premise that severe VL is caused by an exaggerated inflammatory response from the host and that this response is at least partially conditioned by virulence factors of *L. infantum*, we aimed to determine whether a marker of the host's inflammatory response – IL-6 – could be used as a pathway to identify pro-inflammatory virulence factors. To pursue this, we decided to conduct a GWAS on *L. infantum* isolates derived from individuals diagnosed with VL whose plasma IL-6 concentrations were measured.

## Materials and methods

### Participants

At the Natan Portela Institute of Tropical Diseases (IDTNP), a reference hospital for infectious diseases, 66 individuals diagnosed with visceral leishmaniasis were treated. The participants were selected based on diagnostic criteria, specifically those with confirmed VL diagnosis through culture, with parasites frozen in liquid nitrogen. Clinical and laboratory data were meticulously collected from the patient's medical records. For each isolate, a 250 μL aliquot of serum was taken before the initiation of treatment and stored at −20°C to preserve its immunological integrity for subsequent analyses.

### Isolates

The parasites were obtained through bone marrow aspiration and cultured in 2 mL of combined solid and liquid phase medium, consisting of NNN (Neal, Novy, Nicolle) medium and Schneider Insect Medium. After reaching the logarithmic phase of growth, they were cryopreserved in liquid nitrogen (registered in SISGEN-C8035C5) until use.

### Interleukin-6 quantification

A 25 μL aliquot of serum was used for the quantification of the IL-6 cytokine. The experiment was conducted using the BD™ Cytometric Bead Array (CBA) Human IL-6 Enhanced Sensitivity Flex Set kit, on a FACS CANTO 2 flow cytometer (Becton Dickinson, New Jersey, USA).

### Statistical analysis of interleukin-6 concentration in participants

Data on IL-6 concentration and clinical outcomes of the subjects were used to construct a non-parametric receiver operating characteristic (ROC) curve, particularly applied when data do not follow a specific parametric distribution. This analysis aimed to assess IL-6's ability to classify disease status, whether survival or death. In addition to investigating the points of the curve, the area under the curve (AUC) metric was employed to evaluate the accuracy of the test, providing an estimate of the probability of correct classification of a subject at random. Logistic regression was used to understand and model the relationship between variables, offering probabilities associated with different values of the independent variables.

In the ROC curve analysis, the maximum Youden index was employed to determine an optimal cut-off point for IL-6 concentration in individuals. This approach aimed to balance sensitivity and specificity, contributing to a more precise interpretation of the test's performance. All statistical analyses were performed using Stata 15.1 IC software (StataCorp LLC, College Station, USA).

### Parasite DNA sequencing

#### Sample preparation

After thawing the 66 isolates, the parasites were cultured in NNN (Novy-MacNeal-Nicolle) and Schneider's medium (Insect Medium, Schneider, Sigma, St. Louis, USA), supplemented with foetal bovine serum (10%), urine (2%), 100 U mL^−1^ penicillin and 100 μg mL^−1^ streptomycin (Pen/Strep, Gibco, Grand Island, NY, USA). After a 7-day period and confirmation of parasite viability, a passage was made in 10 mL of supplemented Schneider medium. Upon reaching the exponential phase, approximately 5 days later, the tubes containing the parasites were centrifuged at 3000 rpm for 10 minutes at 4°C, and the resulting pellet was washed three times with physiological solution (0.9% NaCl). After the final wash, the parasites were resuspended in 200 *μ*L of 0.9% NaCl and subjected to DNA extraction.

#### Parasite DNA extraction

DNA extraction was performed using the Mini Kit Genomic DNA Purelink (Invitrogen™) with 200 μL of the solution containing the parasites. This procedure is based on the use of specific buffers and proteins for cell lysis, followed by the binding of DNA to the chaotropic salt membrane present in the kit columns. Afterward, the DNA was washed and eluted. DNA quantification was carried out using the Qubit® 2.0 Fluorometer and NanoDrop™ 2000/2000c spectrophotometers, where the concentration and purity of the samples were assessed by the 260 nm/280 nm ratio, respectively. The integrity of the extracted DNA was verified by 1% agarose gel electrophoresis.

#### DNA sequencing of isolates

The DNA from the isolates was sequenced by Macrogen, Inc. using the Illumina® Next Generation Sequencing (NGS) platform, with the HiSeq2500 sequencer and the TruSeq DNA PCR-Free Library Prep Kit.

#### Whole genome sequencing (WGS) analysis with MegaBOLT

The 66 genomes were assembled and analyzed using MegaBOLT v.2.4.0 (MGI), a self-developed bioinformatics analysis accelerator focused on Next-Generation Sequencing (NGS). This software provides comprehensive support for whole genome sequencing (WGS) analysis, from FASTQ data input to generating results in binary alignment map (BAM) format, following alignment, and in Variant Call Format (VCF), derived from variant calling.

The MegaBOLT workflow includes various stages, such as quality control (FastQ/DataQC), read mapping (SAM), position sorting, duplicate removal, marking and base quality score recalibration (BQSR), along with specific modules for variant calling, following GATK's best practices for preprocessing data in variant discovery (Danecek *et al*., 2011). This process includes alignment with the *L. infantum* reference genome (MCAN/ES/98/LLM-724; González-de *et al*., 2017), as well as several data-cleaning steps to correct technical biases and ensure that the data are suitable for analysis, with the final output stored in VCF files containing information about the sequence variations of the analysed genomes (Li *et al*., 2023).

#### Quality control and genome-wide association study (GWAS)

To ensure the quality and integrity of the study, rigorous quality control (QC) was implemented on the genotypic data before performing the genome-wide association study (GWAS) analysis. The VCF file was filtered with a minimum Variant Allele Frequency (VAF) of 20% and a minimum read depth (DP) of 10 to ensure the quality and reliability of the identified variants. Of the 7 recommended quality control steps (Marees *et al*., 2018), 5 were considered applicable to this study: missing data per individual and per SNV, minimum allele frequency (MAF), Hardy–Weinberg equilibrium deviations (HWE), heterozygosity rate and population stratification.

GWAS was conducted to investigate the relationship between SNVs and IL-6 quantifications in 62 isolates of *L. infantum* from patients with visceral leishmaniasis (VL). Although *Leishmania* is an aneuploid organism, the genotypic data were converted to diploid format to standardize the analysis and apply exclusion criteria for polymorphic sites. Regions with more than one variant were removed, ensuring that only single genetic variants were included for greater consistency in the results. The association analysis was conducted using a logistic regression model with binary data, implemented in PLINK software (version 1.9) (Chang *et al*., 2015). In logistic regression, to correct for multiple testing, the ‘–indep-pairwise 50 5 0.2’ parameter was used in PLINK to perform SNV pruning and identify independent variants. R software (version 4.4.1) with the qqman package (version 0.1.9) was used for global visualization of the results, generating a Manhattan plot, Quantile-Quantile (Q-Q) plot and a plot illustrating the distribution of SNV counts per chromosome.

The annotation of the variants regarding the genomic region and functional impact was performed using SnpEff v4.0 (Cingolani *et al*., 2012), with a database created from Tritryp (Shanmugasundram *et al*., 2023) using the *L. infantum* genome version (MCAN/ES/98/LLM-724; González-de *et al*., 2017). This process involved configuring and indexing the genomic data. For the description of genomic regions, data from TriTrypDB and TrypsNetDB were consulted, using the corresponding gene ID (Gazestani *et al*., 2017; Shanmugasundram *et al*., 2023).

## Results

### Characterization and description of the study population

The study population consisted of 50 men (75.8%) and 16 women (24.2%), with an average age of 25.8 years. Among the participants, 27 (40.9%) were children: 4 children under 12 months (6%), 7 (11%) children aged 1 to 23 months, and 16 (24%) aged 2 to 15 years. The remaining subjects were between 16 and 40 years old, with 20 (30%) in this range, while 19 individuals were older than 40 years (29%). A total of 13 subjects (19.69%) presented co-infection with HIV. Of the 66 participants included in the study, 46 survived (70.70%), while 20 died (30.30%). Among the symptoms manifested by the studied population, the most prevalent were splenomegaly (100%), fever (90.90%), oedema (40.90%), vomiting (37.87%), dyspnoea (24.24%), jaundice (19.69%) and sepsis syndrome (15.15%) (Table 1).

**Table 1.** Characterization of the study patients (*n* = 66)

Characteristic	Number (%)
Sex	
Male	50 (75.8)
Female	16 (24.2)
Age group	
<12 months	4 (6)
12–23 months	7 (11)
2–15 years	16 (24)
16–40 years	20 (30)
>40 years	19 (29)
HIV co-infection	
Reactive	13 (19.69)
Non-reactive	53 (80.31)
Clinical outcome	
Survivors	46 (70.70)
Deaths	20 (30.30)
Main symptoms	
Splenomegaly	66 (100)
Fever	60 (90.90)
Oedema	62 (40.90)
Vomiting	25 (37.87)
Dyspnoea	16 (24.24)
Jaundice	13 (19.69)
Sepsis syndrome	10 (15.15)

### Interleukin-6 concentration and epidemiological data

The median IL-6 concentration in the serum of participants with VL was 100.90 pg mL^−1^ (interquartile range (IQR): 32.51; 281.69), with an overall median of 70.72 pg mL^−1^ (IQR: 32.265; 275.848). In the group of subjects who succumbed, the median IL-6 concentration was 170.33 pg mL^−1^ (IQR: 43.37; 609.76), while in surviving individuals, it was 57.8 pg mL^−1^ (IQR: 30.07; 235.35). Additionally, the median IL-6 concentration in participants co-infected with HIV was lower, at 31.77 pg mL^−1^ (IQR: 11.61; 77.39), compared to those without co-infection, whose median was 100.9 pg mL^−1^ (IQR: 35.29; 287.52), *P* value 0.0156. Women had a higher median IL-6 concentration compared to men (343.36 pg mL^−1^ *vs* 60.31 pg mL^−1^, *P* value 0.067) (Table 2).

**Table 2.** Distribution of IL-6 concentration in the serum of participants with VL by age group, sex, HIV coinfection and clinical outcome

Characteristic	Number (%)	Mean (pg mL^−1^)	s.d. (pg mL^−1^)	Median (pg mL^−1^)	Interquartile range (pg mL^−1^)	
					Q1	Q3
Age*						
<12 months	4 (6)	2106.0	3307.4	120.5	36.5	6161.1
12–23 months	7 (11)	411.2	503.7	294.6	41.4	391.7
2–15 years	16 (24)	458.4	159.06	109.1	52.6	675.2
16–40 years	20 (30)	144.5	49.6	49.9	16.3	154.2
>40 years	19 (29)	118.2	32.4	47.0	21.8	159.4
Sex**						
Male	50 (76)	173.6	42.0	60.3	23.0	206.8
Female	16 (24)	943.1	502.2	246.4	52.6	775.3
HIV***						
Reactive	13 (19,7)	123.3	64.7	31.7	11.6	77.40
Non-reactive	53 (80,3)	410.7	1154.4	100.9	35.29	287.52
Outcome****						
Deceased	20 (30)	791.1	1814.7	170.3	43.3	609.7
Survivors	46 (70)	172.8	303	57.8	30.7	235.3
Total	66 (100)					

*P = 0.043; **P = 0.067; ***P = 0.0156; ****P = 0.0281.

Logistic regression was used to understand and model the relationship between IL-6 levels (log-transformed) and the variables age, sex, HIV co-infection and death. The model was statistically significant (LR *χ*^2^ (4) = 19.79, *P* value = 0.0005), with a pseudo *R*^2^ of 0.2444, indicating that the variables explain approximately 24% of the variation in the probability of death. Among the variables analysed, death was significantly associated with IL-6 levels (coefficient = 1.0446, *P* = 0.015), suggesting that higher IL-6 levels increase the likelihood of death in patients with visceral leishmaniasis. Age showed a negative coefficient (coef = −0.0146, *P* = 0.081), indicating that although there is a trend of lower IL-6 levels with increasing age, this association was not statistically significant. Similarly, sex showed a positive association with IL-6 levels (coef = 0.7775, *P* = 0.080), although this relationship did not reach statistical significance. HIV co-infection showed no significant association with IL-6 levels (coef = −0.7479, *P* = 0.114) (Table 3). These results indicate that IL-6 concentration is strongly associated with the death outcome in patients with visceral leishmaniasis, supporting the hypothesis that inflammation, as measured by IL-6, plays a crucial role in the prognosis of the disease.

**Table 3.** Logistic regression results modelling the relationship between IL-6 levels and clinical variables in patients with visceral leishmaniasis

Variables	Coefficient	SE	95%CI	*P* value
Age	−0.0146	0.0083	−0.0309–0.0017	0.081
Sex	0.7775	0.4441	−0.0929–1.6479	0.080
HIV co-infection	−0.7479	0.4732	−1.6754–0.1796	0.114
Death	1.0446	0.4294	0.2028–1.8863	0.015

*IL-6 values are log-transformed.

**Figure 1.**
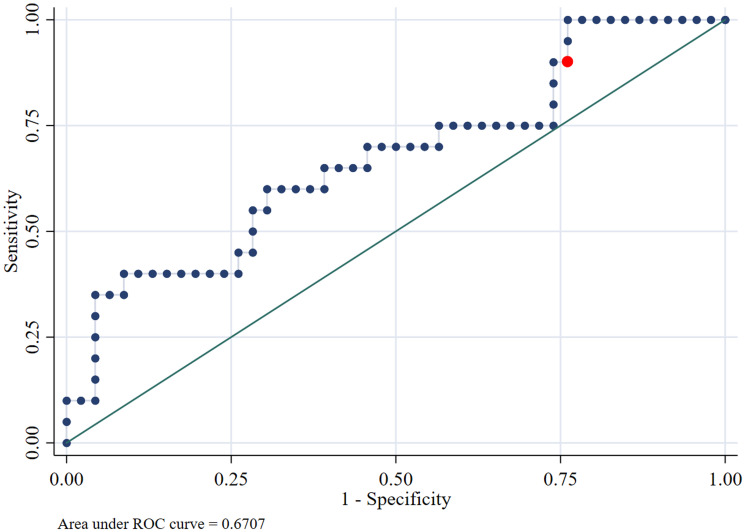
Relationship between IL-6 concentration and mortality outcome: area under the curve analysis with ideal cut-off point. The red point denotes the cut-off point (391.7 pg mL^−1^) that maximizes the correlation between IL-6 concentration and the mortality outcome, highlighting its relevance as a predictor.

An analysis of IL-6 concentration data was conducted in relation to the outcome of survival or death using a non-parametric ROC curve. An area under the curve (AUC) of 0.67 (95% CI 0.52–0.81) was obtained. Subsequently, logistic regression and the Youden Max technique were applied to determine the optimal cut-off point that maximizes the correlation between IL-6 concentration and the mortality outcome. The resulting value was 391.7 pg mL^−1^ (Fig. 1).

### Sequencing and assembly

**Figure 2.**
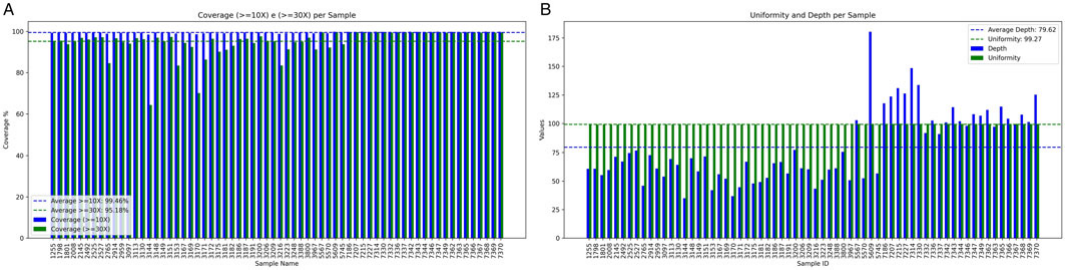
Analysis of genomic coverage and sequencing depth in the samples. Panel 2A shows the genomic coverage at ≥10X and ≥30X for the samples analyzed in the study, illustrating the distribution of genomic coverage across different sequencing depths. Panel 2B presents the analysis of sequencing uniformity and depth per sample, highlighting the consistency of sequencing depth and uniformity across the samples.

Whole-genome sequence data were generated using the Illumina Next-Generation Sequencing (NGS) platform. The sequences resulted in a genomic coverage of at least 10X, with an average of 99.45% (±0.31) and a median of 99.45%, and a minimum coverage of 30X, with an average of 95.17% (±6.24) and a median of 96.55%, mapped against the *L. infantum* JPCM5 reference genome (MCAN/ES/98/LLM-724; González-de *et al*., 2017) (Fig. 2A). The sequences had an average depth of 79.62X and a uniformity of 99.27% (Fig. 2B).

### Genomic variation

**Figure 3.**
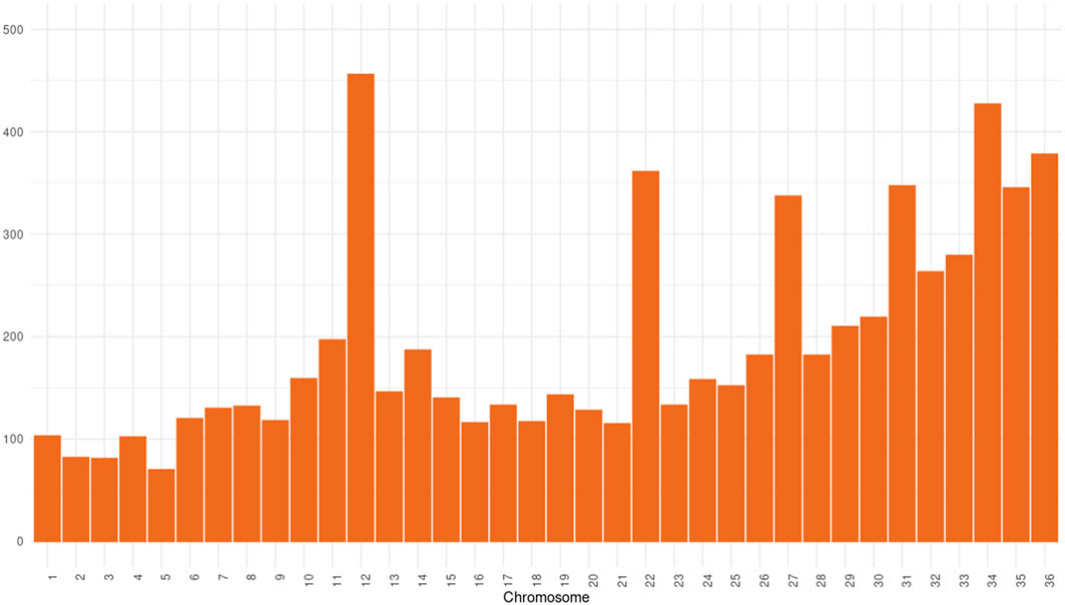
Distribution of the number of SNVs by chromosome. The figure illustrates how the SNVs are distributed across chromosomes, providing an overview of the genetic variation in the study.

In the 66 analyzed genomes of *L. infantum*, 10 029 SNV variants were identified in the VCF file. After applying the filters of VAF ≥20% and DP ≥10, 62 genomes remained, with a total of 6,948 identified SNVs (Fig. 3). Of these, 6,341 were located in protein-coding regions, with 4,999 having a modifier impact, 866 a moderate impact, 454 a low impact and 22 a high impact. Additionally, among the 6,948 SNVs, 5,751 exhibited a minor allele frequency (MAF) of ≤5%.

### Relationship between single nucleotide variants and interleukin-6 concentration levels

**Figure 4.**
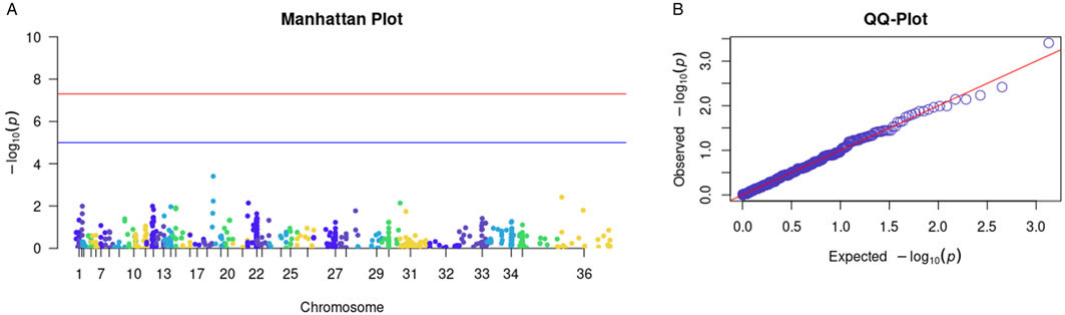
Comprehensive visualization of SNVs. (A) Represents the Manhattan plot, while (B) corresponds to the *Q*–*Q* plot. Both provide an overall visualization of all SNVs identified in the logistic regression, allowing a clear analysis of the distribution and association of the SNVs.

After conducting the association test on 62 samples with IL-6 dosage using PLINK software, we identified 722 variants associated with the logistic regression analysis, using the dichotomized IL-6 concentration values above and below the cutoff point of 391.7 pg mL^−1^. The selection of SNVs was based on different *P* values (See Table 4 in the supplemental material). Figures 4A and 4B display all the SNVs resulting from the logistic regression, including the Manhattan plot and Q–Q plots.

In total, 35 SNVs showed a significant association with IL-6 levels in the logistic regression analysis, all having an odds ratio of 3.3 or higher. Among these SNVs, the majority are located in coding regions, with some associated with hypothetical proteins, along with 3 SNVs found in intergenic regions.

## Discussion

The sample in this study reflected the expected trend of a predominance of male individuals, who represented 75.8% of the cases. Studies conducted in endemic regions have already indicated a higher incidence of cases among men (Andrade *et al*., 2020; Cavalcante *et al*., 2020). However, despite this predominance, the concentration of IL-6 was significantly higher in women. Previous studies have shown that women have higher levels of IL-6 compared to men (Panagi *et al*., 2019; Mun *et al*., 2020). Evidence suggests that gender and sex hormones directly influence the incidence, prevalence and mortality of various diseases, including leishmaniasis, as well as impacting clinical manifestations and responses to established treatments (Lockard *et al*., 2019; de Araújo Albuquerque *et al*., 2021).

The concentration of IL-6 was higher in participants who were HIV non-reactive. Elevated levels of IL-6 have been observed in individuals with active VL, being associated with the severity of cases and the risk of death (Costa *et al*., 2013; Guedes *et al*., 2022). Moreover, in cases of coinfection with VL, IL-6 may play a crucial role as a contributing factor to fatal outcomes (Costa *et al*., 2013).

The dysregulation of the inflammatory response, which is closely associated with disease progression, significantly contributes to the development of systemic inflammatory syndrome (Costa *et al*., 2010; Ribeiro *et al*., 2020). The definition of the cut-off point was based on a prediction analysis of IL-6 for mortality, using the ROC curve and the maximum Youden index, which presented an AUC of 67.1. This analysis indicated that, in our studied population, a concentration of 391.70 pg mL^−1^ may predict mortality in individuals with VL. The association analysis between polymorphisms and IL-6 aimed not to correlate IL-6 concentration with mortality but rather to associate the SNVs with IL-6 concentration. Studies conducted by Costa *et al*. (2013) and Dos Santos *et al*. (2016) demonstrated a strong positive correlation between IL-6 levels and manifestations of severity in VL. Notably, individuals with severe VL had significantly elevated levels of IL-6, with concentrations above 200 pg mL^−1^ being strongly associated with fatal outcomes. These results are consistent with the proximity between elevated IL-6 levels and mortality observed in our study. In healthy adults, the IL-6 level is 1.79 ± 2.03 pg mL^−1^ (Yoshida *et al*., 2002), while in healthy new-borns, this level is 9.8 pg mL^−1^ (Martin, Olander, and Norman, 2001). In another study, IL-6 levels were below the detection limit in over 90% of the blood donors tested (Kildey *et al*., 2014).

GWAS analyses aim to identify SNVs whose frequencies change systematically based on specific phenotypic characteristics (Marees *et al*., 2018). The approach adopted to investigate the relationship between SNVs and IL-6 concentration in patients with VL utilized logistic regression, where the concentration of IL-6 was previously categorized for analysis. Logistic regression is often used due to its flexibility compared to other methods; it allows for consideration of confounding effects by incorporating covariates and is useful for adjusting for population stratification (Rentería *et al*., 2013). In the association analysis, logistic regression obtained 35 SNVs with highly significant *P* values. The *Q*–*Q* plot indicated that this association is a robust assumption, as the points align almost linearly.

It was observed that 23 SNVs identified in the logistic regression analysis are located in regions associated with hypothetical proteins. Among the 8,548 proteins described in *L. infantum* (JPCM5), 40.5% are hypothetical proteins, 3,980 are disordered proteins and approximately 37% are situated in regions with characteristics of intrinsic disorder (Avelar *et al*., 2020). Three SNVs were identified in genes of uncharacterized proteins. In addition to 3 intergenic regions, the remaining SNVs are located in genes encoding amastin surface glycoprotein, kinesin K39, kinase protein and phospholipase C-like protein.

One SNV associated with the kinesin K39 region exhibited a synonymous effect with low impact. Kinesin K39 is a highly antigenic motor protein in *L. infantum*, located in the cytosol of both the promastigote and amastigote forms of the parasite (Gerald; Coppens; Dwyer, 2007). In our analysis, one SNV was identified in the coding region of the amastin protein, with a downstream effect and modifying impact. The gene family of amastin proteins consists of up to 45 members, which perform distinct or complementary functions (Rochette *et al*., 2005). Some of these proteins are involved in the intracellular survival of *Leishmania spp.* in infected hosts, facilitating the transport of ions, metals and nutrients to the internalized parasites (Wu *et al*., 2000; Rochette *et al*., 2005). Amastins have been suggested as potential virulence factors, as they are associated with the intracellular survival of the parasite (Dupé *et al*., 2014). Additionally, these proteins have been found to be highly expressed in *L. donovani* isolated from individuals with VL (Salotra *et al*., 2006).

One SNV exhibited an upstream effect and a modifying impact, with an odds ratio of 8.2, in the gene that encodes the phospholipase C protein. Phospholipases catalyze the cleavage of phospholipid molecules and are involved in various physiological processes, such as cell membrane remodelling, lipid-mediated signal transduction, cell proliferation and virulence (Flammersfeld *et al*., 2018). Three SNVs were identified in uncharacterized proteins, including the DUF3535 protein. Its ortholog in *L. major* (LmjF.36.3340) has been described in stationary promastigotes as a probable signal peptide (Casanova *et al*., 2015). Additionally, the ortholog in *L. major* (LmjF.20.0700), which is a putative ubiquitin-like protein, is described as one of the 25 proteins with differential expression (upstream and downstream) in response to the overexpression of Maf1 (Rivera-Rivas *et al*., 2024). The study by Rivera-Rivas *et al*. (2024) suggests that Maf1 is involved in various functions in *L. major*, including global transcription regulation, cell cycle control, ribosomal biogenesis, lipid metabolism, carbohydrate metabolism and cytoskeletal modification.

A comprehensive understanding of the development, proliferation, virulence and biological processes of an organism is often limited by a lack of knowledge regarding the function of certain proteins. Therefore, the annotation of hypothetical proteins is essential for deepening our understanding of the organism (Folador *et al*., 2018). Virulence is a multifaceted phenotype influenced by various characteristics of pathogens and hosts (Gerstein *et al*., 2019). Understanding the virulence factors of the infectious agent, as well as the immunological mechanisms and host immune response, is crucial for determining the progression and clinical outcome of an infection. The absence of a detailed analysis of the interactions between these elements, also considering environmental conditions, may create gaps in the understanding of virulence. To enhance knowledge about the biology of the parasite and its interactions with the host, it is necessary to identify, characterize and validate new therapeutic targets (Roberts, 2011). One limitation of this study was the absence of environmental covariates, such as sex, age and HIV, in the association analyses. Including these factors could provide a more detailed understanding of how environmental variables interact with genetic characteristics, enriching the interpretation of the results. However, even with the inclusion of these variables, the confirmation of the identified associations depends on independent validation in additional populations (Hayes, 2013).

Furthermore, although the study provided valuable insights with the available sample, the relatively modest sample size (66 samples) may have influenced the statistical power of the analyses. Replication of the results in larger cohorts would contribute to a more robust validation of the identified genetic associations. Although the identified genetic associations suggest potential candidates for functional investigations, genome-wide association studies do not have the capacity to directly predict phenotypes (Marigorta *et al*., 2018). Therefore, further investigations are needed to explore the functional impact of these variants. Additionally, the absence of IL-6 values for healthy controls in the study sample is a limitation, and as a result, comparisons were made with previously published data. This should be considered when interpreting the findings.

This study provided valuable insights into the relationship between genetic polymorphisms and IL-6 levels in patients with VL. The identification of 35 novel variants associated with IL-6 concentration underscores the complexity of the inflammatory response and its connection to disease severity. The prevalence of hypothetical proteins among the identified SNVs highlights the need for further studies to elucidate their biological functions and role in the virulence of *L. infantum*. Additionally, although the sample size is limited, a trend was observed linking elevated IL-6 levels to unfavourable clinical outcomes, emphasizing the relevance of IL-6 as a potential prognostic marker in cases of visceral leishmaniasis. A deeper understanding of these factors could not only improve therapeutic approaches but also inform prevention and control strategies for the disease in vulnerable populations.

## Supporting information

da Silva et al. supplementary material da Silva et al. supplementary material

## Data Availability

The data that support the findings of this study are available from the corresponding author, AMS, upon reasonable request and at Leishmaniasis Research Laboratory, Federal University of Piauí, Piauí, Brazil.

## Supplementary material

The supplementary material for this article can be found at https://doi.org/10.1017/S0031182024001598.
