# Supplementary material for: Genome-wide association study analysis of single nucleotide variants in L. infantum associated with IL-6 inflammatory response in visceral leishmaniasis
Source: Parasitology. 2024 Dec 13;152(8):798–806. doi: 10.1017/S0031182024001598 (PMC12644933; doi:10.1017/S0031182024001598)
Supplement: da Silva et al. supplementary material [file S0031182024001598sup001.docx]

Table 4 – Single nucleotide variants (SNVs) with the strongest association in logistic regression analysis between dichotomous IL-6, considering statistical significance (p < 0.05)

| CHR (LR) | Gene  (LINF_) | Position | SNV (LinJ.) | Change | | Effect | Impact | Annotation | Odds ratio | p-value |
| --- | --- | --- | --- | --- | --- | --- | --- | --- | --- | --- |
| 812952.1 | 190008500 | 132613 | 19.132613 | A/C | upstream | | modifier | Kinase protein (putative) | 16,1 | 0,000393 |
| 812969.1 | 360013100 | 267005 | 36.267005 | G/C | upstream | | modifier | Hypothetical protein (conserved) | 11,19 | 0,003831 |
| 812952.1 | 190008500 | 132619 | 19.132619 | A/C | upstream | | modifier | Kinase protein (putative) | 7,333 | 0,005855 |
| 812955.1 | 220013300 | 343893 | 22.343893 | T/A | downstream | | modifier | Hypothetical protein (conserved) | 8,214 | 0,007227 |
| 812963.1 | 300034800 | 1074583 | 30.1074583 | A/G | upstream | | modifier | Phospolipase c-like protein | 8,214 | 0,007227 |
| 812935.1 | 020011100 | 297523 | 02.297523 | A/G | upstream | | modifier | Hypothetical protein (conserved) | 6,143 | 0,01021 |
| 812945.1 | 120013300 | 403818 | 12.403818 | G/A | upstream | | modifier | Hypothetical protein (conserved) | 6,143 | 0,01021 |
| 812947.1 | 140017500 | 517154 | 14.517154 | G/A | synonymous | | low | Kinesin K39 (putative) | 3,489 | 0,01087 |
| 812948.1 | 150009800 | 164532 | 15.164532 | C/G | synonymous | | low | Hypothetical protein | 4,332 | 0,01218 |
| 812948.1 | 150009800 | 164539 | 15.164539 | G/A | missense | | moderate | Hyphothetical protein | 6,429 | 0,01331 |
| 812948.1 | 150009800 | 164542 | 15.164542 | G/C | missense | | moderate | Hyphothetical protein | 6,429 | 0,01331 |
| 812945.1 | 120014900 | 456201 | 12.456201 | C/G | upstream | | modifier | Hypothetical protein (conserved) | 3,373 | 0,01482 |
| 812969.1 | 360041900 | 1362041 | 36.1362041 | C/G | upstream | | modifier | Protein of uncharacterised function DUF3535 | 7,833 | 0,01596 |
| 812961.1 | 280034600 | 1071945 | 28.1071945 | T/G | upstream | | modifier | Uncharacterised ACR COG1678 (putative) | 5,25 | 0,01693 |
| 812964.1 | 310013700 | 296716 | 31.296716 | T/A | upstream | | modifier | Hypothetical protein (conserved) | 4,964 | 0,01809 |
| 812952.1 | 190007900 | 108805 | 19.108805 | C/A | upstream | | modifier | Hyphothetical protein | 4,174 | 0,0221 |
| 812935.1 | 020011100 | 294525 | 02.294525 | A/C | upstream | | modifier | Hypothetical protein (conserved) | 5,238 | 0,02322 |
| 812955.1 | 220023100- 220023200 | 770938 | 22.770938 | A/G | intergenic | | modifier | Phosphoinositide specific phospholipase C (putative) - Hypothetical protein | 5,238 | 0,02322 |
| 812947.1 | 140010200 | 175369 | 14.175369 | T/G | downstream | | modifier | Amastin surface glycoprotein (putativa) | 5,75 | 0,02962 |
| 812955.1 | 220012400 | 305769 | 22.305769 | G/A | synonymous | | low | Hypothetical protein (conserved) | 5,75 | 0,02962 |
| 812945.1 | 120014400 | 447661 | 12.447661 | A/G | missense | | moderate | Hypothetical protein (conserved) | 4,082 | 0,03597 |
| 812945.1 | 120014400 | 447666 | 12.447666 | A/G | synonymous | | low | Hypothetical protein (conserved) | 4,082 | 0,03597 |
| 812945.1 | 120014400 | 447668 | 12.447668 | T/A | missense | | moderate | Hypothetical protein (conserved) | 4,082 | 0,03597 |
| 812945.1 | 120014400 | 447674 | 12.447674 | A/C | missense | | moderate | Hypothetical protein (conserved) | 4,082 | 0,03597 |
| 812945.1 | 120014400 | 447681 | 12.447681 | C/G | missense | | moderate | Hypothetical protein (conserved) | 4,082 | 0,03597 |
| 812955.1 | 220023200 | 779603 | 22.779603 | G/T | upstream | | modifier | Hypothetical protein | 4,388 | 0,03819 |
| 812955.1 | 220023200 | 779606 | 22.779606 | C/G | upstream | | modifier | Hypothetical protein | 4,388 | 0,03819 |
| 812955.1 | 220023200 | 779609 | 22.779609 | C/G | upstream | | modifier | Hypothetical protein | 4,388 | 0,03819 |
| 812966.1 | 330038500- 330038600 | 1277593 | 33.1277593 | C/G | intergenic | | modifier | Hypothetical protein (conserved) - Hypothetical protein (conserved) | 9,37 | 0,0387 |
| 812955.1 | 220023200 | 777156 | 22.777156 | T/C | downstream | | modifier | Hypothetical protein | 5,417 | 0,0405 |
| 812943.1 | 100005800 | 35677 | 10.35677 | G/C | upstream | | modifier | Dehydrogenase like protein | 4,0 | 0,04059 |
| 812934.1 | 010013400-CHR_END | 273623 | 01.273623 | C/T | intergenic | | modifier | Peptidyl dipeptidase (putative) | 8,643 | 0,04636 |
| 812953.1 | 200012200 | 249762 | 20.249762 | C/T | upstream | | modifier | Hypothetical protein (conserved) | 4,835 | 0,04754 |
| 812953.1 | 200012300 | 279209 | 20.279209 | G/A | downstream | | modifier | Conserved protein uncharacterised function | 4,835 | 0,04754 |
| 812956.1 | 230023900 | 710847 | 23.710847 | C/A | upstream | | modifier | Hypothetical protein | 4,835 | 0,04754 |
